# Brain correlates of subjective freedom of choice

**DOI:** 10.1016/j.concog.2013.08.011

**Published:** 2013-12

**Authors:** Elisa Filevich, Patricia Vanneste, Marcel Brass, Wim Fias, Patrick Haggard, Simone Kühn

**Affiliations:** a Institute of Cognitive Neuroscience, University College London, London WC1N 3AR, UK; b Department of Experimental Psychology and Ghent Institute for Functional and Metabolic Imaging, Ghent University, Gent, Belgium; c Max Planck Institute for Human Development, Center for Lifespan Psychology, Lentzeallee 94, 14195 Berlin, Germany

**Keywords:** Volition, Introspection, Free choice, fMRI

## Abstract

The subjective feeling of free choice is an important feature of human experience. Experimental tasks have typically studied free choice by contrasting free and instructed selection of response alternatives. These tasks have been criticised, and it remains unclear how they relate to the subjective feeling of freely choosing. We replicated previous findings of the fMRI correlates of free choice, defined objectively. We introduced a novel task in which participants could experience and report a graded sense of free choice. BOLD responses for conditions subjectively experienced as free identified a postcentral area distinct from the areas typically considered to be involved in free action. Thus, the brain correlates of subjective feeling of free action were not directly related to any established brain correlates of objectively-defined free action. Our results call into question traditional assumptions about the relation between subjective experience of choosing and activity in the brain’s so-called voluntary motor areas.

## 1 Introduction

Debates over whether humans have the capacity to make free choices have been ongoing for several centuries (Libet, 1999; Thomas, 1894; Wegner, 2002). In contrast, there is wider consensus about the existence of a *subjective experience* of acting freely (Sarkissian et al., 2010). We often have the impression that our internal conscious decisions drive our behaviour. In other words, we feel that our decisions and actions are not simple determined by the immediate environment, but rather expressions of our “agentic self” (Kane, 2005; Schüür & Haggard, 2011).

According to one view, these subjective experiences are illusory (Wegner, 2002), and the “conscious will” is merely a retrospective inference, rather than a direct readout of brain activity associated with action selection or action generation. The experimental studies supporting this view largely used behavioural methods to identify “illusions of will” (Wegner & Wheatley, 1999), and to manipulate their intensity (Vohs & Schooler, 2008). Further support for this view comes from electrophysiological and neurophysiological recordings (Libet, Gleason, Wright, & Pearl, 1983; Soon, Brass, Heinze, & Haynes, 2008). These studies show that the onset of the neural events associated with free decisions precede the reported onset of the awareness of intention, and suggest that conscious intention cannot be a causal factor for free decisions. Interestingly, however, few studies have investigated where in the brain this alleged illusion arises.

Neuroscientific studies have often defined volition by drawing a distinction between the information-processing underlying free and instructed actions. Because this definition makes no appeal to subjective experience, it has proved particularly useful in studies with animals (Thaler, Chen, Nixon, Stern, & Passingham, 1995). In this operationalization, instructed actions are fixed, yet possibly arbitrary, associations of stimuli with movements. Typically in instructed actions, both the timing and type of action are explicitly specified (the *when* and *what* of action (Brass & Haggard, 2008)). In contrast, free actions leave either the “what” or the “when” dimension of action underspecified (“when” (Libet et al., 1983; Thaler et al., 1995), “what” (Krieghoff, Brass, Prinz, & Waszak, 2009; Waszak et al., 2005)). Thus, free actions are those which do not entirely depend on explicit external signals, but depend more strongly on hypothesised internal sources. However, specifying precisely what these internal sources actually are has proved difficult and controversial (Nachev, Kennard, & Husain, 2009; Schüür & Haggard, 2011). They may include action goals (Gollwitzer, 1996), or memories (Tanji, 2001). As such, free actions cannot, by definition, be strongly related to any *single* environmental event, although they may be weakly related to a wide range of environmental inputs (Schüür & Haggard, 2011).

Support for the free vs. instructed account of volition comes partly from primate neuroanatomical evidence (Goldberg, 1985; Passingham, 2007). Lateral portions of the premotor cortices are responsible for instructed or reactive actions driven by explicit external signals, while medial premotor areas, notably the supplementary motor area (SMA), are responsible for free, self-generated or “projectional” action. For example, Thaler et al. (1995) showed that removal of the medial premotor cortex in monkeys impaired their ability to make learnt arbitrary arm movements at their own pace, in order to receive a food reward. The same monkeys showed less impairment to make the same movement following an external auditory cue.

This account of free choice has been supported by several human neuroimaging studies. The contrast between free and instructed movement choices has been consistently associated with increased BOLD signal in the SMA and preSMA, the rostral cingulate zone (RCZ) and the dorsolateral prefrontal cortex (DLPFC) (Cunnington, Windischberger, Robinson, & Moser, 2006; Lau, Rogers, Haggard, & Passingham, 2004; Lau, Rogers, & Passingham, 2006), (see Krieghoff, Waszak, Prinz, and Brass (2011) for a review). In particular, Mueller, Brass, Waszak, and Prinz (2007) have suggested that RCZ is mainly involved in selecting the “what” component, in the context of a given task (Desmet, Fias, Hartstra, & Brass, 2011); whilst preSMA participates in selecting the “when” component of actions. In addition, Thimm, Weidner, Fink, and Sturm (2012) have recently shown that the BOLD activations generally associated with free choice need not be associated with overt movement. Covert unrestricted choices between objects also elicited comparable BOLD activations to free selection of motor responses, centred on the medial frontal cortex regions.

This tradition has therefore capitalised on objective experimental design factors which allow instructed and free choice to be defined in terms of information that is provided by external cues, or is not so provided. This definition bypasses the subjective experience of free choice. However, understanding the neural basis of the common feeling of acting freely is important. On the one hand, if free choice is indeed illusory, understanding the mechanism underlying illusions has long been a productive approach in psychological research. On the other hand, if free choice is not illusory then the neural bases of the subjective experience of free choice may be relevant to understanding how and where free choices occur in the brain.

The scientific tradition reviewed above operationalizes voluntary action based on objective criteria. This tradition implicitly assumes that voluntary action operationalized in this objective way captures the subjective feeling of acting intentionally. However, this important implicit assumption has never been appropriately validated. If the assumption is correct, and there is a correspondence between objective and subjective accounts of free action, then the neural correlates of free and instructed choices defined objectively should roughly match with those defined subjectively. We therefore devised an experimental task in which actions were defined either on the basis of an objective definition, or on the basis of subjective experience. We then investigated the brain correlates associated with free choices under each of the two possible definitions.

Here we use and extend the classic distinction between instructed and free choice to investigate the neural correlates of *subjective* voluntariness. Importantly, we also consider that external guidance can come in varying degrees; so that the instructed/free distinction is not a simple dichotomy between two exclusive categories, but rather represents two extremes of a continuum. On this view, generating an action can involve both internal and external factors. For example, when a driver brakes or accelerates in response to a traffic signal, they are responding both to the external event of the signal’s colour, but their action also depends on an arbitrary rule that they have acquired, and chosen to follow. For our purposes, we consider freedom of choice as a *graded* measure of how independent an action is from an external stimulus. When an action is strongly determined by an external stimulus, it will be “less free” than when it is not. We may then ask whether people subjectively experience degrees of voluntariness underlying individual action decisions, and whether this graded experience originates from graded levels of activation in particular brain areas.

We have adapted the classic task of random number generation (Jahanshahi, Dirnberger, Fuller, & Frith, 2000) to allow both an objective, graded continuum of integration of stimulus information, and also a graded continuum of subjective experience of voluntariness regarding action choice. Random number generation tasks have been used before in relation to volition, but for rather different reasons from ours. In particular, many free selection studies involve asking participants to produce balanced numbers of responses, while avoiding obvious patterns such as alternation. These tasks have been interpreted as covertly asking participants to generate apparently random response sequences (Roepstorff & Frith, 2004). For example, human positron emission tomography (PET) studies during random number generation tasks have suggested a critical involvement of the left dorsolateral prefrontal cortex (DLPFC), the anterior cingulate cortex, the bilateral superior parietal cortex, and the right inferior frontal cortex (Daniels, Witt, Wolff, Jansen, & Deuschl, 2003; Jahanshahi et al., 1995). These areas partially overlap with those identified with free selection tasks (Jahanshahi et al., 1995). In our case, a modified random generation task offered a convenient vehicle to allow participants to experience and report a graded sense of stimulus-independence or freedom of action.

Crucially, in two separate analyses, we compared extreme situations of free and instructed action as operationalized by the classical objective paradigms, and also situations in which actions subjectively *felt* more free or *felt* more constrained, based on self-report. In this way, we hoped to establish the relationship between free actions as traditionally defined, and the subjective experience of having chosen freely.

In the crucial condition for studying graded voluntariness, we presented a number sequence and asked participants to complete the sequence with a number that would make the sequence “look random”. The method that participants used to achieve this presumably varied from person to person, depending on their subjective concept of a random sequence. However, the actual interpretation of “random appearance” was not our central interest here. In contrast we focussed on participants’ subjective feeling of voluntariness associated with whatever choices they had in fact made. Subjective, quantitative report of voluntariness allows us to study the feeling of free choice with high ecological validity. Thus, instructed actions allow for no flexibility of behaviour, whereas free actions, in the everyday sense, may be the result of an internal evaluation of the applicability and relative importance of the several different internal rules.

## 2 Methods

### 2.1 Participants

Twenty-three healthy participants took part in the study (5 female; mean age 22 ± 2 years). All participants gave written informed consent. Procedures were approved by the local ethical committee, and were in accordance with the Declaration of Helsinki. All participants had normal or corrected-to-normal vision. No participant had a history of neurological, major medical, or psychiatric disorder.

### 2.2 Stimuli and procedure

Each participant made manual actions to choose numbers on a screen using a trackball in experimental trials within three different contexts in a random number generation task.

**Fig. 1:**
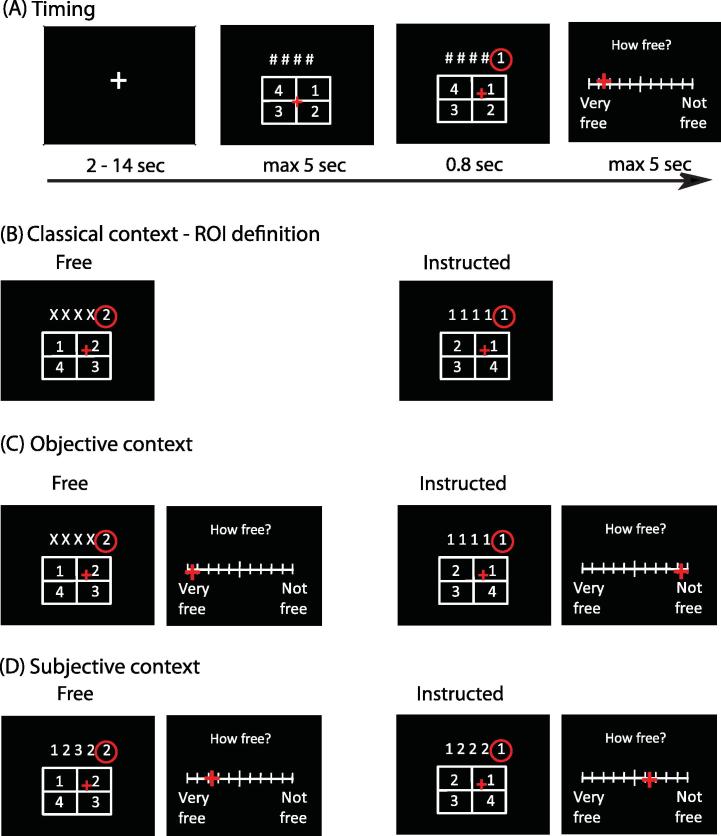
Experimental paradigm. (A) General timing of events. Four numbers or four Xs were displayed on the screen (in the figure, # represents either a number or an X. The symbol # was never actually displayed in the experiment). Participants chose a number by clicking on it with a trackball. Participants were then asked to click on a visual analogue scale to rate how free their choice had been. (B) The classical context was used to identify regions of interest associated with the contrast free > instructed in independent data. In the objective context (C) trials were defined as free or instructed *a priori*. In objective free trials, a series of four X’s was displayed and participants were free to choose any number from the response grid. In objective instructed trials, participants saw a sequence of four identical numbers (“1 1 1 1” in this example) and participants were asked to complete the sequence by clicking on the same number that was displayed. Participants then indicated their feeling of voluntariness. In the subjective context (D) four numbers were displayed, and participants were asked to complete the sequence with the fifth number “in order to keep the sequence looking random” (see Section 2 for details). Trials were then classified *a posteriori* according to a median split of each participant’s subjective reports.

Each trial proceeded as follows (see Fig. 1). A white fixation cross was displayed on a black background. The fixation duration was sampled from a pseudologarithmic distribution, and ranged from 2 to 14 s. The fixation cross served as a variable period constituting an implicit baseline BOLD measure. Immediately after, either a sequence of four numbers (from 1 to 4) or four X’s appeared on the screen, above a 2 × 2 response grid. The position of each number in the grid was randomly assigned and changed in every trial. Participants held an MRI-compatible trackball on their lap. The mouse cursor was displayed on the screen using a red “+” sign, initially positioned on the centre of the response grid. Participants were instructed to select a number from the response grid by moving the mouse cursor to the chosen number and clicking on it. The choice of number was based in the number stem presented (see below). Once the number was selected, it was displayed next to the stem for 0.8 s.

In two of the contexts the question “How free was your choice?” (“Hoe VRIJ voelde je keuze aan?” in Dutch) was displayed above a visual analogue scale (VAS). The VAS had 10 subdivisions, and its extremes were labelled “Very free” and “Not free” (“HEEL vrij” and “NIET vrij”). The left–right orientation of the VAS labels was counterbalanced across participants, but kept constant for each participant throughout the whole practice and experimental sessions, to avoid confusion. Participants were asked to indicate how free they felt their choice had been by clicking with the cursor on the appropriate position. Participants were reminded that they could use the whole range of the VAS.

The maximum response time for the number selection and the voluntariness rating was 5 s. If participants had given no response after this time had elapsed, a message appeared and the next trial started.

First, we compared conditions of free choice versus instructed choice in a *classical* context (see below), operationally defined in the same way as the classical literature on free/instructed choices. Second, in an *objective* context, participants performed the free and instructed conditions again, but each trial was followed by a rating of how free they felt their immediately-preceding action choice had been. Finally, in the crucial *subjective context*, participants chose which of several actions to make following presentation of a suggestive stimulus, and rated on a continuous scale the extent to which their action choice had been free or not free, with respect to the information given in the suggestive stimulus.

The subjective context necessarily involved a subjective rating, whilst the classical context did not, making it difficult to compare these conditions directly. The objective context was designed to get round this problem, by providing a condition which was informationally equivalent to the classical context, but also included the element of introspective report. In this way, BOLD activity associated with the objective context could be contrasted with activity in the subjective context because both contexts included both an action selection and a judgement event in each trial.

#### 2.2.1 Classical context

With the classical context, we aimed at identifying patterns of BOLD signal associated with actions that have been classically treated as free. In free trials, the presented sequence was always “X X X X”. In these trials, participants were free to choose any of the four numbers (1, 2, 3 or 4) displayed on screen as their action. In instructed trials, the sequence contained one single number repeated four times (e.g., “1 1 1 1”). In these trials, participants were instructed to choose the number that was displayed on the screen (i.e., 1).

The *classical* context was effectively used as a localizer, to define regions of interest (ROIs). The BOLD activity for the other two contexts was analysed for ROIs identified independently from the data from the classical context.

#### 2.2.2 Objective context

Our main aim was to compare the brain correlates of the extreme free and instructed conditions with those of a subjective context in which participants could themselves report how free their choice had been by means of a VAS (see below). In order to make the two contexts comparable, we included a VAS rating in the objective context. In this way, any BOLD activity differences between the objective and the subjective contexts could not be attributed to the mere presence of the VAS.

#### 2.2.3 Subjective context

With the subjective context, we aimed at providing participants with a scenario in which they could experience a graded sense of more or less free choice. Unlike in the other two contexts, the numerical stimulus presented in each trial contained a pseudorandomized sequence of numbers. Participants were instructed to use this sequence as a suggestive guide for their response. They were asked to choose a number that would make the stimulus sequence “look random”. We assumed that folk knowledge would guide participants in their choices (Nickerson, 2002). Each participant might have felt highly constrained by some preceding sequences, and very unconstrained by other sequences. Participants were asked to report choices as being less and more free in those trials respectively. The beliefs and strategies would differ from person to person, and were not relevant for our purposes. Instead, we used random sequence generation as a means to provide participants with a graded and reportable experience of voluntariness, in the sense of freedom from constraint. We could then use this to investigate the brain activity associated with the experience of voluntariness.

The numerical stimulus therefore served to prompt the next action choice to some extent. The extent to which it did so was assumed to influence the experience of subjective freedom of choice. Any of a number of rules could relate the numerical stimulus to the chosen action. We chose to ask them to make the stimulus “look random”, and to report their subjective feeling of freedom of choice. The precise *form* of the completion rule used may have varied across participants and was irrelevant to our purposes. Instead, we were interested in the *extent* of constraint provided by the numerical stimulus and the rule, which participants were asked to report. We assumed that subjective reports of freedom of choice were informative of this relative constraint. In this way, voluntariness was not directly manipulated experimentally. Instead, introspection was taken as a reliable method to report subjective experience.

It is important to emphasise that, unlike previous studies (e.g., van Eimeren et al., 2006) here we did not *assume* that the subjective feelings of voluntariness would directly correlate with the number of available alternatives. Instead, we gave participants a complex rule and allowed them to interpret it. In that way, we hoped to distil the bases of the feelings of voluntariness, *independently* from any preconceptions from the experimenter’s part.

The sequences for the subjective context were generated by a pseudorandomized procedure. We defined a measure of “stimulus space” for each sequence as the number of different numbers present in the stem (irrespective of position). The sequences “3 3 3 3”; “2 2 1 2”; “3 1 4 4” and “2 3 4 1” are examples of sequences with stimulus spaces of 1, 2, 3 and 4 respectively. We distributed the total 160 subjective trials into 4 blocks of 40 trials. Pilot results suggested that participants’ ratings of subjective freedom of choice were related to the size of stimulus space. Therefore, each block contained sequences with equal numbers with all possible stimulus spaces. The sequences for the subjective context were the same for all participants, to allow for potential comparisons across participants. Their order of appearance was randomized across blocks and trials.

Additionally in the subjective context, a memory question was displayed every 10 trials. A four-number sequence was presented and participants were asked if that sequence had been presented in the preceding 10 trials, with a maximum response time of 5 s. This memory question was aimed at encouraging participants to pay attention to the number stimuli presented on every trial, and the responses were not analysed.

Each participant performed two consecutive blocks of 40 trials in each of the classical and objective contexts. They also performed 4 consecutive blocks of 40 trials in the subjective context, which formed the key focus of the study. The order of the contexts was randomized across participants.

Before scanning, participants were trained with at least one practice block for each context, always in the same order: classical; objective, subjective. Training continued until participants felt comfortable with the task. The experiment in the scanner lasted approximately 70 min.

After scanning, participants completed five personality questionnaires addressing feelings of control (Rotter, 1967) belief in free will (the free will and determinism scale (Rakos, Laurene, Skala, & Slane, 2008); the social desirability scale (Crowne & Marlowe, 1960); and two self-control questionnaires (Rosenbaum, 1980; Tangney, Baumeister, & Boone, 2004)). In addition participants completed a semi-structured questionnaire about the strategies they had adopted in the completion of the random number sequences.

### 2.3 fMRI data acquisition

Participants were positioned head first and supine in the magnet bore. Images were collected with a 3T Trio MRI scanner system (Siemens Medical Systems, Erlangen, Germany), using an 8-channel radiofrequency head coil. First, 176 high-resolution anatomical images were acquired using a T1-weighted 3D MPRAGE sequence [TR = 2500 ms, TE = 2.58 ms, image matrix = 256 × 256, FOV = 220 mm, flip angle = 7°, slice thickness = 0.90 mm, voxel size = 0.9 mm × 0.86 mm × 0.86 mm (resized to 1 mm × 1 mm × 1 mm)]. Whole brain functional images were collected using a T2^*^-weighted EPI sequence, sensitive to BOLD contrast (TR = 2000 ms, TE = 35 ms, image matrix = 64 × 64, FOV = 224 mm, flip angle = 80°, slice thickness = 3.0 mm, distance factor = 17%, voxel size 3.5 mm × 3.5 mm × 3 mm, 30 axial slices). A varying number of images were acquired per run owing to the self-paced initiation of trials.

### 2.4 Data processing and analysis

Trials with reaction times (RTs) (for either the number choice or the voluntariness rating) shorter than 0.2 s or longer than 5 s were discarded from the analysis. Instructed trials with incorrect responses were also discarded.

Trials from the classical and objective contexts were classified into “free” and “instructed” according to the stimulus presented in each trial: e.g., “1 1 1 1”, “2 2 2 2” etc. were classified as instructed, and “X X X X” was classified as free. In contrast, trials from the subjective context were classified into “feels free” and “feels instructed” by means of a median split on the distribution of each participants’ subjective reports. The median for each participant was calculated on the basis of all the valid trials (>0.2 s and <5 s of reaction time – RT) across all four subjective context blocks. Thus, the meaning of “free” and “instructed” is not identical across contexts, so the design is not fully factorial. Rather, our interest focussed on assessing whether the different ways of defining free and instructed in the different contexts would be based on qualitatively different neural structures.

The fMRI data were analysed with statistical parametric mapping, using the SPM8 software (Wellcome Trust Centre for Neuroimaging, University College London, London, UK). The first four scans of all EPI series were excluded from the analysis to minimise T1 relaxation artefacts. A mean image for all scan volumes was created, to which individual volumes were spatially realigned by rigid body transformation. The high resolution structural image was coregistered with the mean image of the EPI series. The structural image was normalised to the Montreal Neurological Institute template. The normalisation parameters were then applied to the EPI images to ensure an anatomically informed normalisation. A commonly applied filter of 8 mm FWHM (full-width at half maximum) was used. The time series data at each voxel were processed using a high-pass filter with a cut-off of 128 s to remove low-frequency drifts. The subject-level statistical analyses were performed using the general linear model. The events were defined as the onset time of the stem and response grid. Movement times were also included as parametric regressors to account for variance associated with simple motor activations. All resulting vectors were convolved with the canonical haemodynamic response function (HRF) and its temporal derivative to form the main regressors in the design matrix (the regression model). Realignment parameters in all 6 dimensions were also entered in the model to account for variance associated with head motion. The statistical parameter estimates were computed separately for each voxel for all columns in the design matrix. Contrast images were constructed from each individual to compare the relevant parameter estimates for the regressors containing the canonical HRF. The group-level random effects analysis was then performed. The resulting maps were thresholded with *p *< .001 and cluster-size corrected by means of Monte Carlo simulation. Accordingly significant effects were reported when the volume of the cluster was greater than the Monte Carlo simulation determined minimum cluster size volume (25 voxels), above which the probability of type I error was below 0.05 (Cox, 1996).

## 3 Results

### 3.1 Behavioural results

Voluntariness ratings varied widely both within and between participants. To investigate variation between participants, we calculated the median of each participant’s voluntariness ratings across all blocks. This median had a mean value across participants of 4.05 (±1.68 SD). Variation within participants was considered as a function of free vs instructed trials. Trials in the classical and objective contexts were classified as free or instructed *a priori*; whilst trials in the subjective context were classified *a posteriori*, on the basis of the participants’ subjective reports. To validate the design and analysis, we examined the overall distribution (for all participants) of the ratings of voluntariness for the objective free and instructed conditions separately. Overall, the proportion of free trials (“X X X X” displayed) subjectively classified as instructed was 0.041%, and the proportion of instructed trials (e.g. “2 2 2 2” displayed) subjectively classified as free was 0.043%. This shows that an *a posteriori* classification criterion (according to voluntariness ratings) closely matched the *a priori* classification criterion (according to the information presented in the stimulus). This implies that in the objective condition, participants used the VAS participants as we expected.

**Fig. 2:**
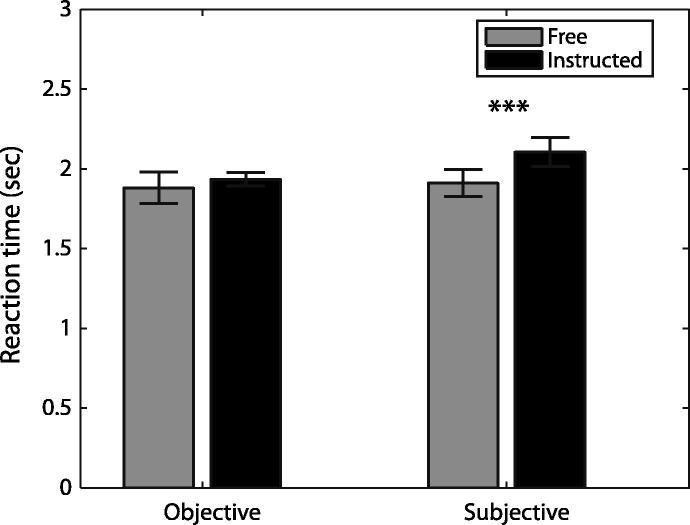
Mean reaction times across participants for all free and instructed trials in the objective and subjective conditions. Subjective instructed conditions were associated with longer reaction times than subjective free conditions (*p *< .001).

To evaluate whether there were any differences in behaviour across conditions, mean RTs were obtained for free and instructed conditions (see Fig. 2). Because the meaning of *free* and *instructed* was not identical in the objective and subjective contexts (see later), a factorial ANOVA was not appropriate. Instead, we compared free and instructed trials in each context, using paired *t*-tests. There were no differences between the RTs for free and instructed trials in the objective context (*t*_22_ = 0.68, *p* = .503). However, instructed trials in the subjective context were consistently associated with longer RTs, as compared to free trials in the subjective context (*t*_22_ = 4.93, *p* < .001). This may reflect the fact that instructed conditions in the subjective context imposed more complex restrictions on the possible choices, and therefore slower selection processes.

#### 3.1.1 Subjective context – behavioural analysis

As an initial approach to the behavioural analysis of the chosen numbers, we examined whether participants were following an exclusion rule for the stem completion task. The reported voluntariness ratings might then simply reflect exclusion behaviour. We classified trials in a binary fashion. Exclusion trials were defined as those in which the number chosen was not included in the presented stimulus space. In contrast, inclusion trials were those in which the number chosen was included in the stimulus space. This analysis could only be computed for trials with at least one number repetition. Results showed that participants predominantly excluded the numbers present in the stimulus space. We computed the exclusion ratio as the proportion of exclusion trials to the total number of valid trials. The mean exclusion ratio was 0.63 ± 0.15 (±SD), and was significantly different from 0.5, which would indicate no preference for exclusion behaviour (*t*_22_ = 4.06, *p* < .001). However, the voluntariness ratings were not solely related to an exclusion vs. inclusion factor. The voluntariness ratings for exclusion trials and inclusion trials were 4.82 ± 1.54 and 4.08 ± 1.6 respectively, and they were not significantly different (*t*_22_ = 1.7, *p* = .1).

**Fig. 3:**
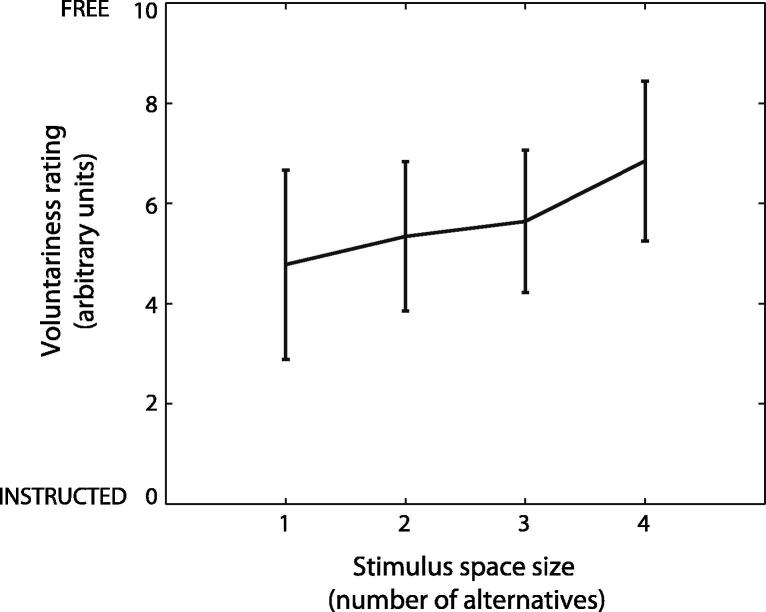
Average across participants of feelings of voluntariness as a function of the size of the stimulus space. Stimulus space is defined as the number of different digits present in the sequence shown. The sequences “3 3 3 3”; “2 2 1 2”; “3 1 4 4” and “2 3 4 1” are examples of sequences with stimulus spaces of 1, 2, 3 and 4 respectively.

To further analyse which factors may have influenced participants’ feelings of voluntariness, we investigated whether the number of different digits in the presented sequence (i.e., the “stimulus space”) might determine the degree of perceived voluntariness. We therefore computed the correlation between the size of the stimulus space and the voluntariness rating for each subject (see Fig. 3). A trend analysis revealed a significant and positive linear relationship *F*(1, 22) = 18.16, *p* < .001) between feeling of voluntariness and stimulus space size. A sequence with only one number represented (e.g., “3 3 3 3”) has a stimulus space of 1. These sequences with small stimulus spaces were associated with responses having the lowest voluntariness ratings, perhaps because participants experienced such stimuli as strongly discouraging a free choice “3” as the response. In contrast, sequences with stimulus spaces of 4 (e.g., “3 2 1 4”) were associated with the highest voluntariness ratings, perhaps because they appeared to offer no particular constraint on participants’ choice. While these suggested explanations of perceived voluntariness as interesting possibilities, they are tangential to our main aim, to investigate the neural correlates of perceived voluntariness.

### 3.2 fMRI results

#### 3.2.1 Classical context, free vs. instructed

We first indentified brain areas showing a differential BOLD activity for the two externally-defined conditions; free and instructed. This contrast served effectively as a functional localizer. The brain areas identified were used to define regions of interest (ROIs) in which BOLD activity for free and instructed trials was analysed for the independent data obtained from the remaining two contexts.

The contrast free > instructed in the classical context revealed increased BOLD signal in RCZ/SMA, bilateral inferior parietal sulcus (IPL) left dorsolateral prefrontal cortex (DLPFC) and left premotor cortex (PMC) (see Table 1). These findings are consistent with existent report of free action > instructed action contrasts (Cunnington et al., 2006; Lau et al., 2004, 2006).

#### 3.2.2 ROI analysis in objective and subjective contexts

The ROIs identified in the free > instructed contrasts in the classical context were tested in independent data from the two other contexts; namely subjective and objective contexts.

**Fig. 4:**
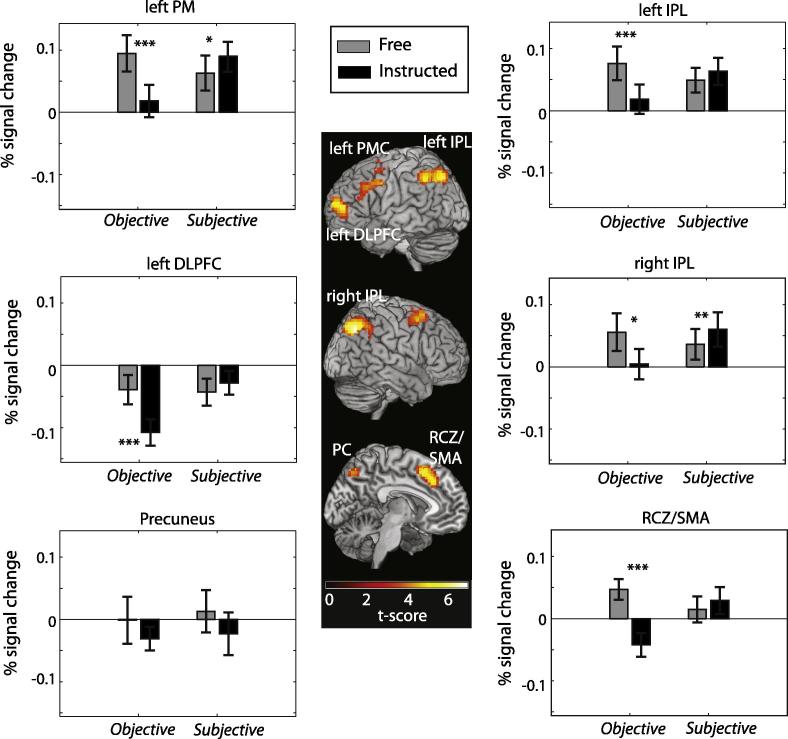
Effect of subjective feeling of free action in brain areas defined by objective freedom of action. Results of the ROI analysis for RCZ/SMA, right and left IPL, left DLPFC and left PMC. ROIs were defined on the basis of the free > instructed contrast in the classical context. Percent signal change from data from two independent datasets were then determined in those ROIs. BOLD activations from the objective context correspond to that found in the classical context. On the contrary, BOLD signal from the subjective context follows an inverse pattern in all but one (the precuneus) of the ROIs identified. Error bars show standard error of the mean. RCZ, rostral cingulate zone; SMA, supplementary motor area; IPL, inferior parietal lobe; DLPFC, dorsolateral prefrontal cortex; PC, precuneus.

Free and instructed conditions could not be taken to be equivalent across the objective and subjective contexts, for two main reasons. First, free and instructed conditions in the subjective case were expected to relate to degrees of voluntariness along a continuum, rather than representing categorically different situations. Second, “noise” due to errors in subjective report might have reduced the strength of the contrast between free and instructed conditions in the subjective context, relative to the objective context. For these reasons, a factorial analysis was not appropriate. Instead, for each separate context, we compared per cent signal change for free and forced conditions. This analysis was done within each of the ROIs identified by the free > instructed contrast in the classical context (see Fig. 4 and Table 2).

The only difference between the classical context, used to identify the ROIs, and the objective context is that the latter also included a VAS judgement. Thus, unsurprisingly, all six ROIs analysed showed increased levels of BOLD activity in the free condition as compared to the instructed conditions in the objective context.

Strikingly however, when this analysis was repeated in the subjective context, the pattern of BOLD activity did not match the one in the classical context (see Fig. 4 and Table 2). Indeed, in five of the six ROIs analysed, the difference in BOLD activity between free and instructed conditions was reversed in the subjective context compared to the objective context. In two ROIs (right IPL and left PM) this reversed pattern was statistically significant. In these areas, BOLD activity was statistically *lower* for actions that had felt free as compared to actions that had felt less free, but was nevertheless statistically higher for actions that were objectively free, as compared to instructed.

#### 3.2.3 Subjective context – whole brain analysis

**Fig. 5:**
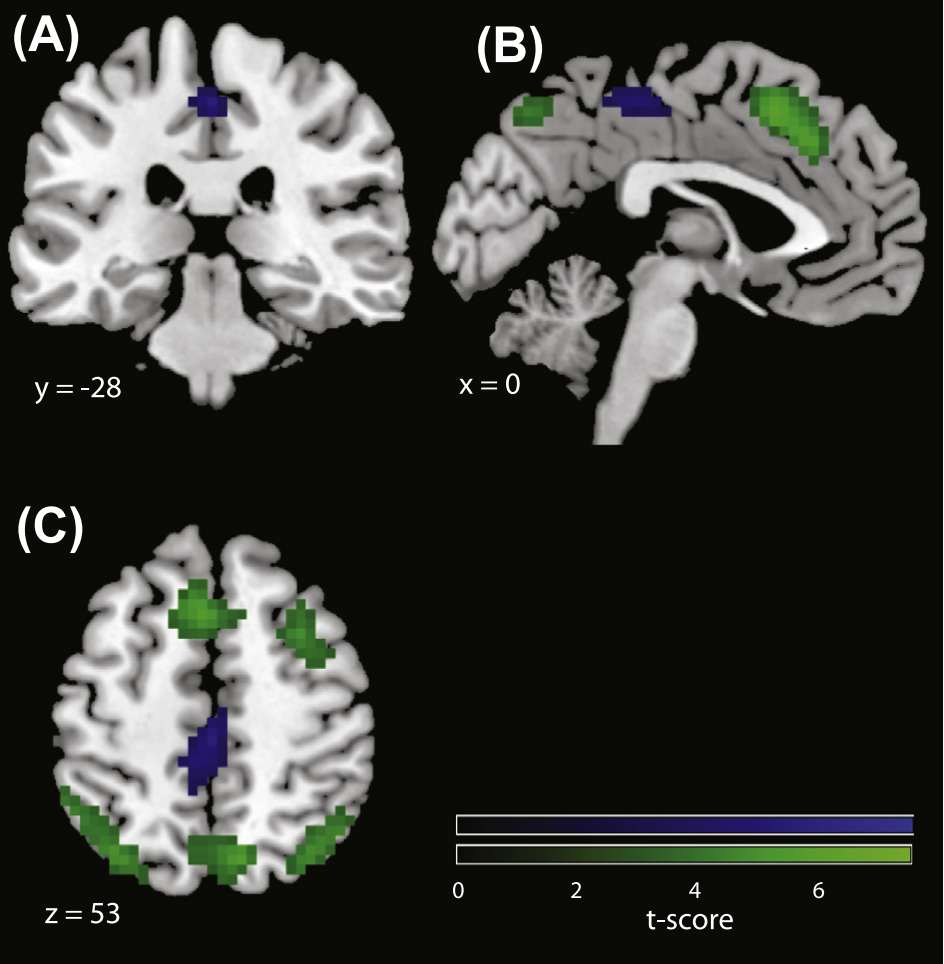
Subjective free > subjective instructed in whole brain (blue) and objective free > objective forced (green). Panels (A–C) show coronal, sagittal and axial planes respectively. Blue: Postcentral region showing increased BOLD signal for the contrast free > instructed in the median split data from the subjective context. BOLD signal peaked at MNI coordinates (*x* = 4 *y* = −21 *z* = 49) and (*x* = 0 *y* = −28 *z* = 53). Results from this contrast in the subjective context are non-overlapping with those from the same contrast in the classical context (green). BOLD activations were corrected for multiple comparisons by means of a Monte Carlo simulation (*p *< .001, minimum cluster size: 25 voxels).

BOLD activity associated with voluntary choice as identified by subjective report did not match with that identified by a classical contrast between free and instructed actions. Specifically, in those areas identified by classical free > instructed choice, we found no evidence for stronger activity when participants felt subjectively more free compared to when they felt subjectively less free. In this sense, objectively and subjectively defined free choice did not overlap. However, no direct inferences about an interaction effect can be made by the simple combination of one significant and one non-significant *t*-test (Nieuwenhuis, Forstmann, & Wagenmakers, 2011). Therefore, to further examine the mechanisms associated with the feeling of voluntariness, and their relationship to the objective operationalization of free action, we did a whole-brain analysis for the contrast free > instructed (median split) in the subjective context controlling for stimulus space and RT by including them as regressors in the first-level model. Only one area, in the medial postcentral region, showed increased BOLD signal for this contrast after whole-brain correction by means of a Monte Carlo simulation (see Fig. 5). BOLD signal showed two peaks of activity in this region, in MNI coordinates (*x* = 4 *y* = −21 *z* = 49) and (*x* = 0 *y* = −28 *z* = 53. This pattern of BOLD activation did not share any commonalities with the pattern found for the contrast free > instructed in the objective context, confirming the results from the ROI analysis.

Finally, we looked for BOLD signal activations that were parametrically modulated by the subjective rating of voluntariness in a whole brain analysis. No areas showed BOLD that survived the correction for multiple comparisons.

## 4 Discussion

Free action has classically been operationalized as action in underdetermined external environments. It has been contrasted to instructed action, in which actions are fully specified by external stimuli. Although this objective operationalization does not make explicit reference to the subjective experience of acting freely, the objective and subjective freedom of action are often implicitly assumed to be related, see for example (Goldberg, Ullman, & Malach, 2008).

We have investigated the relation between the classic operational definitions of free and instructed action and the subjective feeling of acting freely. Participants chose in each trial one out of four response alternatives. We measured BOLD activations in classical conditions in which these free and instructed actions were defined in the objective, classical way, either leaving the response choice to the participant (free trials), or fully specifying the required response in each trial (instructed trials). Our results were in line with previous literature. In particular, we confirmed that medial frontal and parietal BOLD activity is associated with free actions. Results of the comparison between free and instructed conditions showed significantly greater BOLD responses for free compared with instructed in RCZ/SMA, left DLPFC, bilateral IPL and left PMC. These results are consistent with previous reports (Cunnington et al., 2006; Lau et al., 2004, 2006). When participants were additionally asked to rate how free their choices felt, subjective reports in this objective context were consistent with the operational definitions of free and instructed choice, and BOLD contrasts replicated previous studies. This part of our results is broadly consistent with the classical view of voluntary action, and confirms a relation between internal generation of action and the experience of volition (Krieghoff et al., 2011).

We next compared this pattern of BOLD activations with those obtained in a subjective context where participants selected actions according to the combination of a numerical stimulus stem and a completion rule (“look random”). The completion rule aimed at providing each participant with a situation in which they could experience an ecologically valid graded sense of voluntariness.

We assumed that participants might use completion rules to conform to the required “random appearance” of sequences. The precise completion rule (e.g., repetition avoidance, (Ginsburg & Karpiuk, 1994) could vary across participants according to their conceptions of randomness.

Although the completion rule was not considered to be critical in the context of this experiment, we also investigated participants’ number choices, and voluntariness ratings. As an initial approach, we measured the number of exclusion trials (those in which the number chosen was not included in the presented stem). Participants indeed tended to base their number choice on an exclusion strategy. Crucially however, the mean voluntariness ratings did not differ between exclusion trials and inclusion trials, suggesting that participants based their voluntariness ratings on other factors, and that the BOLD contrast between choices subjectively rated as free vs. instructed cannot be simply explained by exclusion-related activity.

Also, and as it was mentioned above, stems with large stimulus spaces (i.e., no number repetitions) did not allow participants to use an exclusion rule, but were at the same time rated most consistently as free. Therefore, the ability to use an exclusion rule (i.e., simply choose the number that is not included in the stem) is unlikely to be the core of feeling free.

However, the actual rules used for “random” generation are not relevant for our analysis, and the requirement to generate random numbers served merely to provide a plausible response space within which some responses might seem more free than others. We considered this subjective experience, independently of the precise completion rule adopted, and of the particular response given. We contrasted trials associated with higher subjective ratings to those with lower subjective ratings of voluntariness.

The neural correlates of the subjective feeling of freedom were quite different from the neural correlates of free choice as classically operationalized. Our ROI analysis revealed that in five of the ROIs identified (ACC, bilateral IPL, left DLPFC and left PMC), BOLD activity in the objective context was higher for free trials as compared with instructed trials. In stark opposition, in the subjective context BOLD activity showed a trend to be lower for free trials in these five ROIs. Only one area, the precuneus, showed the same pattern of BOLD activity in the objective and subjective contexts. Univariate increases in precuneus BOLD activity are not typically associated with voluntary action. Instead, the precuneus has been linked to self-referential tasks and experience of agency (Cavanna & Trimble, 2006). Intriguingly however, multivariate patterns of activity in this area can predict outcomes of free choices (Soon et al., 2008). This may speculatively suggest a hierarchical position for the precuneus in the free generation of response alternatives.

A whole brain fMRI analysis identified a cluster of BOLD activation in a medial postcentral area correlating with the reported subjective feelings of free > instructed choices. This did not overlap with any of the areas identified by the free > instructed contrast in the classical context. Our results suggest that the classic operationalization of free action and the subjective experience of choosing freely are dissociated.

An account of voluntary action based on subjective report revealed a brain area that had not been reported before in this context. The increase in BOLD activity in the medial postcentral cortex correlated with the subjective feeling of freedom in choosing actions, but was not related to the objective informational definition of free action. Therefore, we argue that the feeling of acting freely in naturalistic situations may be independent of the classical free/instructed distinctions.

Our results give rise to three main questions. First, why does the subjective experience of acting freely dissociate from the classical experimental manipulations of free action? Second, what is the functional relevance of the postcentral area found to be related to subjective feelings of acting freely? And finally, what is the significance of subjective report in the study of volition?

### 4.1 Why are objective and subjective contexts dissociated?

To understand why the subjective experience of acting freely does not correspond to the objective operationalization of free action, we examined which responses were reported to be based on free choices.

We compared the mean voluntariness ratings across all possible stimulus set sizes (where the stimulus set size was defined at the number of different numbers present in the stem). Participants judged that sequences with low stimulus spaces (e.g. “3 3 3 3”) were felt more instructed ones, perhaps because a voluntary response to these sequences would have strongly precluded choosing the repeated response (e.g., “3”). In contrast, sequences with stimulus spaces of 4 (e.g., “3 2 1 4”) were associated with the highest voluntariness ratings, perhaps because they appeared to offer no particular constraint on participants’ choice.

This behavioural result interestingly suggests that perceived voluntariness in our task is not related to the number of available alternatives but from how strongly the environment is interpreted as precluding otherwise available alternatives. We do not know the rules or mechanisms that participants used to select actions in the subjective context. Therefore, the cognitive processes in subjective and objective contexts may differ, limiting our ability to compare them. However, we cross-validated perceived voluntariness ratings with the free/instructed conditions in the objective context. The success of this validation suggests that our design would reveal any possible contribution of the brain mechanisms for free choice of action to subjective voluntariness. Since no such contribution was found, we tentatively conclude that subjective voluntariness dissociates from the brain systems that are objectively involved in internal generation of action.

### 4.2 Alternative accounts

Several alternative explanations for the dissociation between objective and subjective context should be considered here. We found differences in the patterns of BOLD activity associated with the free > instructed contrast between the objective and subjective contexts. Clearly, the two contexts differed in more than one respect. For example, the subjective context required a complex evaluation of the number sequence and may have recruited random generation processes (Jahanshahi et al., 2000). In addition, our design included a memory question about the number sequences in the subjective, but not the objective context. However, we argue that the differences in BOLD activity that we identified between objective and subjective contexts cannot be easily explained by differences in task demands. Mental processes such as random number generation would have occurred in *both* free and forced trials within the subjective context, and in *none* of the trials in the objective context. Consequently, the comparison between free and forced trials *within* each context would have subtracted out any potential BOLD activity that was directly related to differential task demands.

Additionally, the differences in patterns of BOLD activations may be related to the nature of the free/instructed distinction in each context. There is a strong and categorical difference between free and instructed trials in the objective context. Conversely, the difference between free and instructed trials in the subjective context is slight and gradual. One might therefore predict smaller differences between free and forced conditions in the subjective context as compared to the objective context. This was not the case. There was a trend in all ROIs identified (except the precuneus) for an *inversion* of the pattern of BOLD activation in the subjective context, as compared with the objective context. This trend became significant in two of the 6 ROIs analysed, namely the right IPL and left PM. More importantly, the free > instructed contrast in the subjective context revealed significant increases in BOLD activity in the postcentral area that were not significant in the objective context.

To account for an inversion in patterns of BOLD activity, it would be necessary to appeal to an implausible inversion of the meaning of the terms “free” and “instructed” on the part of our participants. Therefore, we argue that simple differences in the nature of free and instructed contexts cannot easily account for the results reported here.

Instead, we argue that the subjective feeling of acting freely may be associated with increased BOLD activity in the postcentral area. However, other more speculative explanations cannot be ruled out. For example, activity in the medial postcentral cortex may mediate increased difficulty in finding a random-looking sequence, or other differences related to task processing such as the amount of information that was taken into consideration for the number choice.

Importantly however, previous data do not strongly support these alternative accounts. In particular, increases in task difficulty are typically associated with prefrontal, rather than medial-central, areas (e.g., Barch et al., 1997).

### 4.3 The role of restricted choice on BOLD activity

Trials in the subjective context represent an intermediate situation, between the two objective extremes of free and instructed actions. In other words, even those trials subjectively judged as instructed will require a certain degree of choice.

Two relevant studies have addressed the effect of varying degrees of choice on BOLD activity. Forstmann, Brass, Koch, and Von Cramon (2006) designed a task selection experiment, in which participants were either instructed on which task to perform, or could choose the task between either two or three task alternatives. They found that BOLD activity in RCZ was higher for task choice conditions, but did not vary with the *scope* of choice (i.e., two vs. three possible tasks). This may explain why we did not find BOLD signal increases in the classical areas related to free choice in the subjective context. However, and importantly, limited set of response alternatives cannot readily explain the increase in BOLD activity in the medial postcentral region.

In a previous study (van Eimeren et al., 2006) participants were asked to freely select one out of a number of available response alternatives, ranging from 1 to 4. This design, which did not include a subjective judgement component, allowed the authors to compare conditions of no selection (one available alternative) and restricted selection (between 2 and 3 available alternatives. If the medial postcentral activation is simply due to a limited availability of alternatives, then we should also have found the activation observed in the study by van Eimeren et al. (2006) in the contrast of restricted selection > no selection conditions. Their contrast revealed increased BOLD activity in frontal, premotor and parietal areas, but not in the medial postcentral cortex that we report here. The results by Van Eimeren (2006) do not support the possibility that the BOLD activation in the medial postcentral area is simply related to the limited response space of alternatives.

### 4.4 What is the function of the medial postcentral region?

Could the activation in the medial postcentral area be due to a confound in our task, rather than a true correlate of experience of free choice? Here we examine this possibility.

In tasks requiring free selection, human participants typically hold their previous responses in working memory. Monitoring the contents of working memory can then prevent repetition of behaviour, and can promote generation of seemingly random response sequences (Goldman & Rosvold, 1970). Consequently, working memory acts as a confound that is hard to separate from free selection. Increased working memory load has been associated with increases in BOLD activity in IPL (Rowe, Toni, Josephs, Frackowiak, & Passingham, 2000), but not in DLPFC (Hadland, Rushworth, Passingham, Jahanshahi, & Rothwell, 2001). Given that the objective context led to relatively little cognitive load, it is possible that participants were additionally monitoring their previous history of choices when selecting a response. This would have meant increased working memory function in the objective context. . On the other hand, the more cognitively demanding subjective context may have precluded working memory effects on free trials. This could explain the attenuation of BOLD activity in the ROIs we analysed. However, we found not just attenuation, but an inversion of the free-instructed difference in the subjective compared to the objective context. Working memory accounts cannot easily explain this inversion.

The phenomenology of free action has rarely been addressed experimentally, despite a strong theoretical interest in the issue (Nahmias, Morris, & Nadelhoffer, 2004), but see (Haggard, Cartledge, Dafydd, & Oakley, 2004). The paracentral area that we found to correlate with the experience of free choice has been related to resting state activity (Mazoyer et al., 2001) and default network functions (Mason et al., 2007). However, this area is not generally identified as a core component of the default mode network. Nevertheless, our data remain compatible with the idea that this area may be linked to reflective processes that give rise to the conscious feeling of free will (Goldberg et al., 2008).

### 4.5 What is the value of relying on subjective experience?

Subjective report provides unique access to phenomenal consciousness (Chalmers, 1997), but is notoriously problematic as a guide to neural bases of experience Guggisberg, Dalal, Schnider, and Nagarajan (2011) have recently considered neural correlates of subjective experience related to voluntary action. In an MEG study, the authors investigated the neural substrates of sensory events, of actual movements, and of intentions to move. In addition, they characterised the neural substrates of introspection about the timing of each of these events. They found that the spatial extent of the neural correlates of introspection of events did not match the neural loci of the first order events at which introspection was directed (but see Lau et al. (2004) for the opposite result). Our study mirrors that by Guggisberg et al. (2011) in the sense that the brain areas that underlie introspection about a given process (here the feeling of free choice between action alternatives) do not necessarily match the first order processes to which introspection refers (i.e., choosing between action alternatives).

Several other studies have directly addressed the subjective experience of intending to act. These studies have revealed an involvement of both frontal and parietal areas in the phenomenology of intended action. For example, Fried et al. (1991) found that direct electrical stimulation to medial frontal cortex led to subjective “urges” to act. More recently, Kühn and Haggard (Kühn, Brass, & Haggard, 2012) found that BOLD activity in a caudal portion of SMA correlated with the strength of the “temporal binding” effect, an implicit marker of agency (Haggard, Clark, & Kalogeras, 2002). On the other hand, Desmurget et al. (2009) reported the conscious experience of intending to act following direct stimulation of the parietal cortex. These studies have associated medio-frontal and lateral parietal areas to the feeling of intended action, but not the postcentral area that we report here. One important difference between those studies and ours should be pointed out. In those studies, the timing of action was not specified. Consequently, the subjective experience of action intention may be related to the feeling of impending action. In our experiment, in contrast, an action was to be made at a specific time. Actions could only differ in terms of their freedom of constraint by the external environment. Therefore, the subjective experience reported here did not have to do with the feeling of being about to act, but rather with the feeling of internal generation of one of a range of response alternatives.

Importantly, our results do not undermine the validity of classical operational distinctions between free and instructed choice, nor do they rule out some relation between the operational distinction and the experience of voluntariness. However, our results do suggest that the experience of voluntariness is not simply a consequence of the same brain processes involved in free action selection. Where then, does the experience of free choice arise? There are at least two possibilities. First, the feeling of freely choosing might be confabulated (Wegner, 2002), rather than being a *bona fide* experience at all. Indeed, evidence from choosing between alternative manual responses suggests that the experience of volition is only generated by processes occurring *after* action selection, rather than before (Haggard & Eimer, 1999). On this view, the process of selection itself would be phenomenally silent. Second, the experiences of voluntary choice might arise from a pathway parallel to the (unconscious) pathway that generates action itself (Wegner, 2002).

### 4.6 Conclusion

Our experiment relates to the traditional philosophical question of “free will”. Regardless of whether freedom of will exists or not, most people would agree that they generally feel that *they* choose their own actions (Nichols, 2011). We did not aim to contribute to the debate over whether free will exists. Rather, we tried to investigate the *experience* of free will scientifically. The subjective experience of free choice may be a phenomenal experience similar to other phenomenal experiences, and dependent on a distinct neural substrate (Block, 1995). Our results suggest that this experience may not derive from brain circuits involved in action selection, but from quite different brain circuits.

## Figures and Tables

**Table 1:** Results of whole brain analysis in the classical context (free > instructed). The RCZ/SMA cluster extends to both rostral cingulate zone and supplementary motor area and is ambiguously identified by different toolboxes. RCZ, rostral-cingulate zone; SMA, supplementary motor area; IPL, inferior parietal lobe; DLPFC, dorsolateral prefrontal cortex; PC, precuneus.

Area	Peak coordinates (MNI space)			Peak *z*-score	Cluster size (number of voxels)
	*x*	*y*	*z*		
Right IPL	35	−63	42	5.42	206
Left IPL	−28	−70	46	4.88	217
RCZ/SMA	−4	18	49	4.82	286
Left DLPFC	−35	49	7	4.70	113
Precuneus	11	−70	53	4.62	85
Left PM	−42	4	39	4.20	112

**Table 2:** Results of statistical tests for each region of interest, as identified by the free > instructed localizer, derived from the independent data in the classical context.

Region of interest (peak MNI coordinates)	Pairwise comparisons			
	Free – instructed in objective context		Free – instructed in subjective context	
	*t*	*p*	*t*	*p*
RCZ/SMA (−4 18 49)	7.13	<.001	−1.33	.19
lDLPFC (−35 49 7)	4.54	<.001	−1.23	.23
lIPL (−28 –70 46)	4.83	<.001	−1.02	.32
rIPL (35 –63 42)	3.31	.003	−2.05	.05
lPM (−42 4 39)	3.80	<.001	−2.07	.05
Precuneus (11 –70 53)	–	–	–	–
